# Postoperative Pain Exacerbation After Adenotonsillectomy Due to Oral Candida Infection?

**DOI:** 10.7759/cureus.32115

**Published:** 2022-12-01

**Authors:** Nick S Schofield, Jan Y Quiz, Thomas Schrepfer

**Affiliations:** 1 Otolaryngology, University of Florida, Gainesville, USA

**Keywords:** complication, dehydration, candida, candidiasis, tonsillectomy, adenotonsillectomy

## Abstract

Post-tonsillectomy complications can include bleeding, dehydration, edema, airway obstruction, and infection. Oral candidiasis or thrush is a rare complication that can occur post-operatively. We describe a case of a 10-year-old female with oral candidiasis as a postoperative complication of bilateral adenotonsillectomy, presenting on postoperative day (POD) 7 for poor oral intake secondary to worsening odynophagia. A physical exam revealed an easily scrapable, white plaque located mainly over her surgical sites, tongue, and hard palate. Microscopic examination of tissue scrapings revealed pseudohyphae confirming the diagnosis of oral candidiasis. She was treated with seven days of topical nystatin therapy, including topical and systemic pain control with significant improvement of symptoms by POD 13 and complete resolution on POD 21.

## Introduction

Over 500,000 tonsillectomy cases are performed annually in the United States in children less than 15 years old, making it one of the most common surgical procedures in the pediatric population [1]. Postoperative complications can include bleeding, infection, dehydration, airway obstruction, edema, and velopharyngeal insufficiency (VPI). An oral fungal infection, such as oral candidiasis, is a very rare post-op complication, with no current published data on incidence. To our knowledge, only one case reported in the literature describing local candida infection as a complication following tonsillectomy [2].

Candida species are commonly part of the normal oral flora. Oral carriage of Candida species is generally asymptomatic, occurring in up to 80% of healthy individuals, with a higher prevalence found in healthy children, young adults, and hospitalized patients [3]. Disturbances in the oral flora can cause Candida overgrowth which warrants antifungal treatment. The proper antifungal treatment is based on the clinical presentation, severity of symptoms, and if the underlying immunologic disease is present [4]. Several factors affect the likelihood of developing oral candidiasis: immunologic and endocrine disorders, dietary factors, age, malignant and chronic diseases, hospitalization, smoking history, and hyposalivation [5,6]. The likelihood of a patient developing oral candidiasis without one of the predisposing risk factors is extremely low. Here we describe a 10-year-old female presenting with oral candidiasis manifesting as a postoperative complication of bilateral adenotonsillectomy.

## Case presentation

A 10-year-old female underwent bilateral adenotonsillectomy for treatment of obstructive sleep apnea (OSA). The tonsils were resected with a monopolar cautery and the adenoids were removed via suction cautery. The oropharynx and nasopharynx were then irrigated with normal saline and hemostasis were achieved. Intraoperatively, the patient received 8mg of intravenous (IV) dexamethasone, but no antibiotics. There were no complications noted on the day of surgery. Specimens (tonsils) were not sent for histopathological analysis, since no malignancy was suspected [7].

The patient was reportedly doing well between postoperative day (POD) 1 to 5 when pain medication including ibuprofen 400mg every eight hours and acetaminophen 650mg every six hours were given. However, odynophagia had progressively worsened, and the patient’s mom reported seeing thicker white plaques intraorally on POD six.

**Figure 1:**
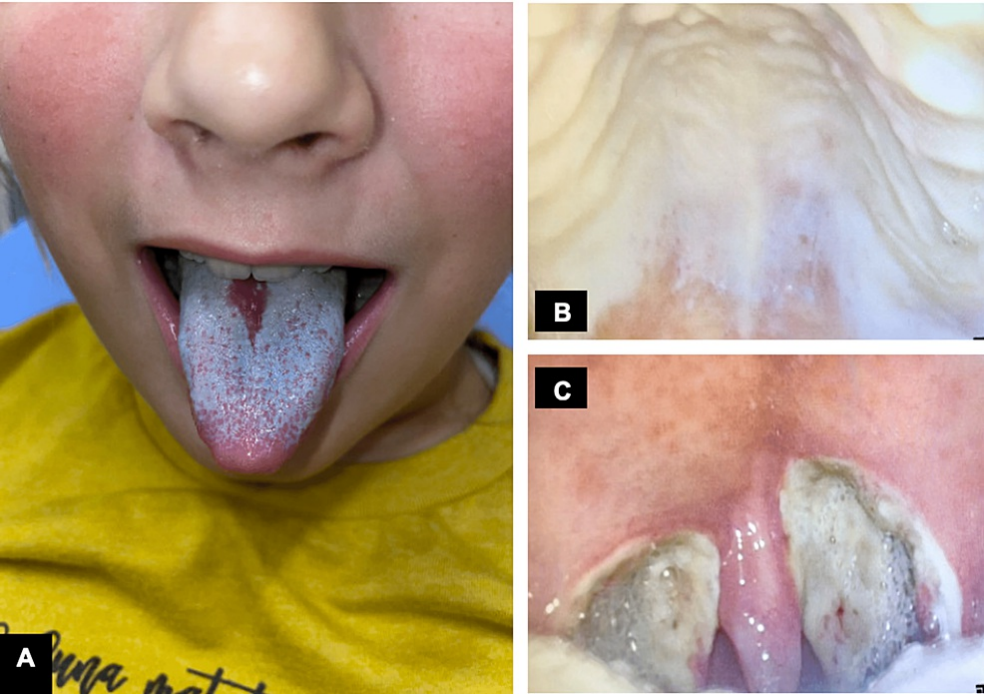
Images taken on postoperative day 7 depicting white plaques on (A) tongue, (B) hard palate and (C) tonsillar fossae respectively.

**Figure 2:**
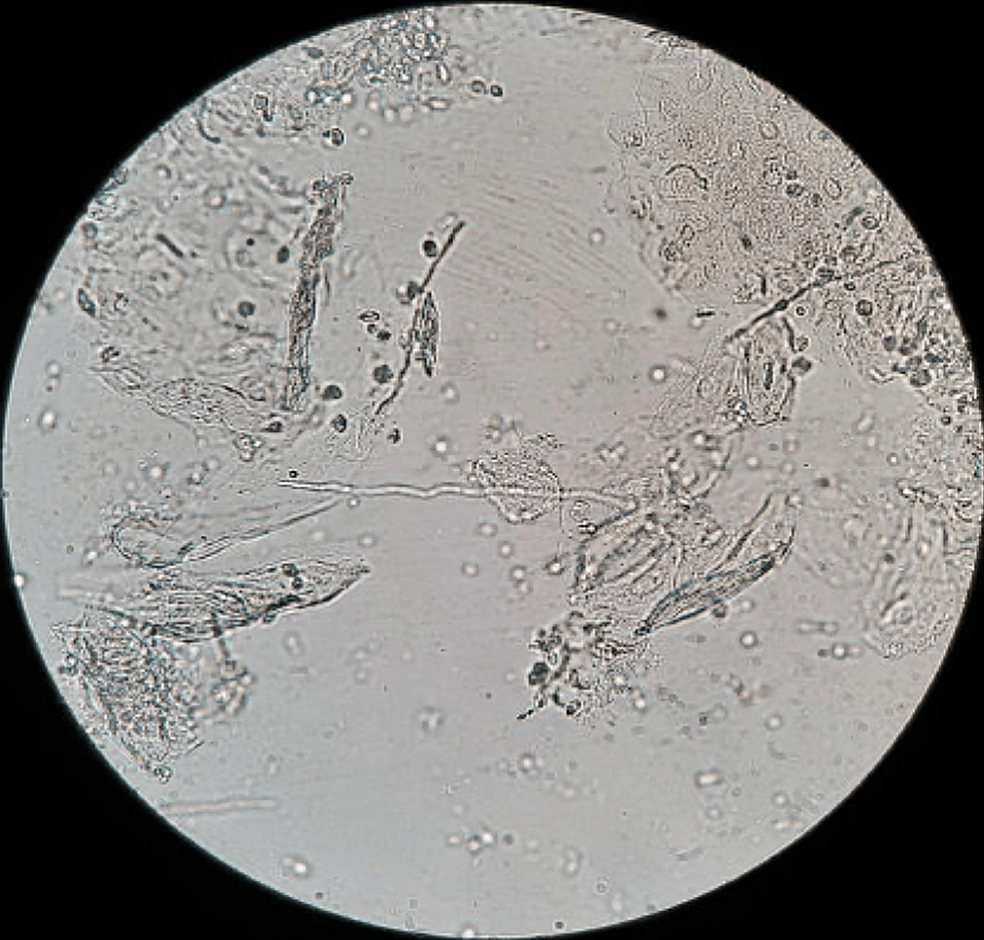
Microscopic evaluation of tissue sample from emergency department (ED) visit depicting pseudohyphae indicating the pathogen to be Candida species.

**Figure 3:**
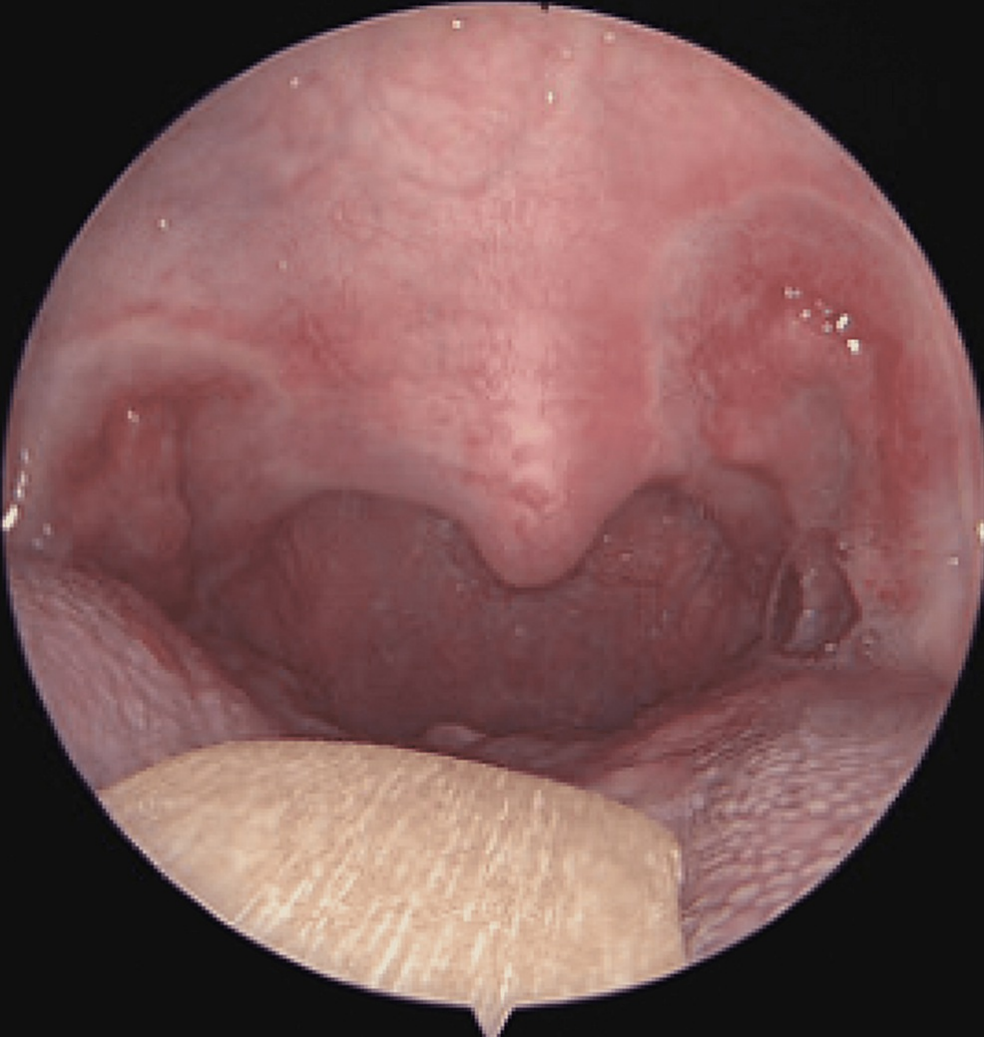
Office visit on postoperative day 21 depicting well-healed tonsillar fossa and no sign of residual Candida infection.

On POD 7, she presented to the Emergency Department (ED) for decreased oral intake secondary to worsening odynophagia that was refractory to acetaminophen and ibuprofen. A physical exam revealed thick, white patches that were easy to scrape, located mainly on the tonsillar fossae, tongue, and hard palate (Figures 1A-1C). Her posterior pharyngeal wall was clear. There was no pathological appearing secretion, mass or lesion noted from her nasopharynx, and her blood work showed normal laboratory values. Microscopic evaluation of the tissue scrapings revealed pseudohyphae confirming the diagnosis of oral candidiasis (Figure 2). She had no previous history of Candida infection. Once she had been hydrated with IV fluids, she was given nystatin mouthwash and instructed to use the medication four times per day for seven days. She also received liquid oxycodone for pain control. The patient was then seen on POD 13 with almost complete resolution of her symptoms and restoration of her appetite/activity level, but white exudate was still noted to be in the tonsillar fossae. She returned on POD 21 without pain or discomfort and with no sign of residual candida infection (Figure 3). Clinically, her OSA symptoms had resolved, and she did not have any signs or symptoms of VPI or nasopharyngeal/oropharyngeal stenosis.

## Discussion

Candida species are fungi that commonly compose part of the normal oral flora in humans. Rozkiewicz et al. found that approximately 40% of healthy school and preschool-age children had Candida albicans as part of their normal flora [8]. The carriage rates increase in the elderly and infants because of decreased immunologic function [9]. These fungi can exist harmlessly as part of the normal oral flora, but if there is a disturbance in the normal flora that causes an overgrowth of Candida species this is termed oral candidiasis and requires antifungal treatment.

There are many risk factors both local and systemic that can predispose a person to develop oral candidiasis. Severely low oral intake secondary to odynophagia in a patient is contributary in two ways. Foremost, oral food and water intake have been shown to decrease the risk of developing oral candidiasis [10]. In addition, this results in dehydration and contributes to hyposalivation and xerostomia. Xerostomia can predispose a patient to develop oral candidiasis because there are many antimicrobial proteins as well as IgA antibodies in saliva which serve to protect against infection [10].

While it is established that topical corticosteroid therapy and prolonged systemic therapy can increase the risk of oral thrush [11,12], the literature review did not show any evidence for increased risk of one-time therapy of IV corticosteroids. At our institution, we routinely administer intraoperative corticosteroids, such as IV dexamethasone (0.5mg/kg, maximum dose of 8 mg) in children undergoing adenotonsillectomy due to its positive effect on postoperative nausea and vomiting, as well as decreased early postoperative pain without increasing risk of postoperative hemorrhage [13,14].

Other local risk factors include denture-wearing, topical corticosteroid therapy, and smoking. Systemic risk factors, which may lead to the disseminated disease include neutropenia, age-related immunosenescence, broad-spectrum IV antibiotics, AIDS-stage HIV, systemic immunocompromise, and nutritional deficiencies [6,15].

There are many different clinical manifestations of oral candidiasis which can be simply classified as acute manifestations, chronic manifestations, or chronic mucocutaneous candidiasis syndromes [6,15]. Our patient presented with acute pseudomembranous candidiasis also known as “thrush.” On physical exam, thrush presents with multifocal, curdy, white plaques throughout the oral mucosa. An important diagnostic factor to distinguish thrush from precancerous/cancerous lesions of the oral cavity/oropharynx is the ability to easily scrape off these white plaques. The plaques themselves are composed of desquamated epithelial and immune cells, as well as yeast and hyphae [6,15].

Candida, like many other microorganisms in the environment, exist in surface-attached biofilms. The oral cavity represents an optimal environment where hyphae and extrapolymeric material contribute to biofilm growth, increasing the adhesive capacity and anti-fungal resistance of Candida [6,16]. The biofilm limits the penetration of substance through the matrix and protects cells from host immune responses. There is a significant association between microorganism capability of biofilm formation and mortality. Therefore, it is crucial for all healthcare professionals to consider the implications of biofilms in recurrent or refractory oral candidosis. Preventive strategies include appropriate oral hygiene with careful mechanical cleaning of teeth and dentures, as well as disinfecting alcohol-free mouthwashes [6].

The diagnosis of oral candidiasis is mainly clinical and the presence of easy-to-scrape white plaques on the oral mucosa is usually enough to establish a diagnosis. If there is any doubt, or if the white plaques recur after treatment, microscopic examination or culture of the tissue scrapings can further support the diagnosis [5,17]. In oral candidiasis, the preparation of specimens using potassium hydroxide (KOH) is the best diagnostic test for the microscopic demonstration of Candida [6,15]. Since Candida species are encountered as normal flora of the mucous membranes, digestive tract, and skin, the presence of (pseudo-) hyphae is necessary for proper diagnosis. Depending on environmental conditions, Candida can undergo morphological changes (i.e., budding yeast cells and filamentous form) [3]. The formation of hyphae or pseudohyphae in human tissues has been interpreted as a sign of virulence correlated with the development of a pathogenic process.

If there is no clear cause for the candida infection, further work-up is warranted to determine existing predisposing risk factors. In our case, the patient, unfortunately, moved away from our area but planned to set up well child visits with a new primary care provider. Follow-up of at least six months after surgery should be considered to rule out residual OSA, VPI, and nasopharyngeal/oropharyngeal stenosis. However, the latter is extremely rare and so far, has not been encountered at our institution [18,19].

Treatment of the infection depends on the severity and whether the patient is immunocompromised. First, any underlying causes for the infection should be dealt with. In our case, poor oral intake and hyposalivation were causative factors. Thus, they were corrected with IV fluids, and subsequent pain medication to increase oral intake of food and water. In cases of mild disease, local antifungals, such as nystatin, can be used as such in our case. For more severe cases with underlying immunocompromised states, systemic treatment with fluconazole is necessary [20].

## Conclusions

Common postoperative complications after tonsillectomy include bleeding, infection, dehydration, airway obstruction, edema, and VPI. Oral candidosis can be a sign of impaired local or systemic defense mechanisms (including reduced saliva secretion), altered oral flora, or poor oral hygiene. In this instance, lack of sufficient pain control in the acute post-operative period likely contributed to persistent odynophagia and subsequent overgrowth of candida. With only one other report in the literature, this case confirms that while very rare, oral candidiasis is a potential postoperative complication of tonsillectomy but is easily managed with topical therapy and pain control.
